# Operating room (OR) requirements

**DOI:** 10.1007/s00068-025-02823-9

**Published:** 2025-03-18

**Authors:** Roman Pfeifer, Frank Hildebrand, Sascha Halvachizadeh

**Affiliations:** 1 Department for Traumatology, University Hospital Zurich, Zurich, Switzerland; 2 Department of Orthopaedics, Trauma and Reconstructive Surgery, RWTH Aachen University, Aachen, Germany

**Keywords:** Polytrauma, White Book, ESTES

## Abstract

The *International Health Facility Guidelines 2023* provide critical recommendations for operating room (OR) design and management, addressing layout, equipment specifications, and safety protocols. By adhering to these guidelines, healthcare facilities can ensure optimal conditions for surgical procedures, minimise risks to patients and staff, and enhance overall efficiency. These recommendations serve as a vital resource for healthcare administrators, architects, and medical personnel, facilitating high-quality surgical care and improved outcomes for trauma patients.

## Introduction

This chapter is based on the *International Health Facility Guidelines 2023*. These guidelines establish minimum acceptable standards for health facilities, bridging the gap between national regulations and international best practices.

Several key factors must be considered when assessing OR requirements for treating trauma patients:

## Institutional and administrative provisions

- Facility briefing and accessibility.
- Mobility and engineering services.
- Environmental design.
- Feasibility planning and cost guidelines.

### Medical provisions

- Infection control measures.
- Equipment planning.
- Staffing qualified healthcare professionals.

Operating units provide a safe, controlled environment for diagnostic and/or surgical procedures under anaesthesia and peri-operative care, including post-procedure recovery. These units, often large theatres, accommodate multidisciplinary teams and vary depending on hospital infrastructure.

## Key considerations for OR design and functionality

### Location and access

- Operating units must be located near the emergency unit, trauma bay, and intensive care unit (ICU) to enable rapid, direct, and discreet transfer of patients. Transport through public corridors should be avoided where possible.
- Corridors must be wide enough to accommodate patient beds, trolleys, or stretchers, along with accompanying personnel and equipment such as respirators, infusion stands, and monitors. Special attention must be paid to door widths for easy passage between units.

## Functional and safety standards

- ORs must accommodate multiple medical specialties simultaneously while maintaining uninterrupted functionality (including electricity).
- Infection control requires:
  - Adherence to hand hygiene, aseptic techniques, and ‘standard precautions’.
  - Positive pressure air-conditioning and HEPA filtration to meet international standards.
  - Antiseptic cleaning protocols facilitated by OR design.
  - Local temperature control for the OR environment.
- Radiation Protection measures must comply with national and international guidelines for radiation shielding, with plans and specifications reviewed by certified physicists or qualified experts.

## Staff safety and protection equipment

Measures to ensure staff safety include:

- Sterile surgical gowns, gloves, and caps.
- Eye and face protection.
- X-ray aprons for radiation safety.
- Guidelines for the disposal of sharps and contaminated waste.
- Restricted access to the OR for unauthorised personnel.

### Minimum equipment for trauma ORs

Trauma patients should be treated in dedicated trauma ORs equipped to address a wide range of potential injuries. Essential equipment is listed below. If a dedicated trauma OR is unavailable, pre-prepared trolleys with essential equipment should be mobilised to the OR in use.

## Diagnostic and surgical equipment

- Essential Tools:
  - Modern X-ray fluoroscopic equipment with dedicated OR radiology technician(s).
  - Interventional hybrid operation capabilities.
  - Radiolucent operating table(s) suitable for trauma management.
  - Fracture table applicable for trauma management.
- Emergency Surgery Equipment:
  - Laparotomy and thoracotomy systems.
  - Chest tubes and surgical airway systems.
  - Neurosurgical intervention tools (e.g., craniotomy, burr holes).
  - External fracture fixation and pelvic reduction systems.
- Definitive Surgery Equipment:
  - Intramedullary nailing systems for long bones.
  - Plating systems (mini, small, large, locked, and standard).
  - Hip fracture fixation plates and nails.
  - (Hemi-) Arthroplasty systems for the hip, knee, and shoulder.
  - Pelvic and acetabular plate systems.
  - Cannulated screw sets (large and small).
  - Power drills, saws, burrs, and reamers.
  - Bone reduction clamp system.
  - Screw removal sets.
  - Arthroscopy systems.

## Conclusion and needs for the future

The future of trauma operating rooms lies in addressing challenges through innovative and collaborative solutions. Advanced technology integration, such as hybrid ORs and evolving surgical tools, holds the potential to enhance both efficiency and outcomes. However, ensuring compatibility and usability requires careful planning and robust training programmes for healthcare professionals.

Emergency preparedness remains critical, necessitating fully equipped ORs and comprehensive protocols for triage, resource allocation, and communication. Staffing skilled surgical teams and providing continuous professional development are equally essential to maintaining high standards of trauma care.

By prioritising these areas and fostering collaboration among healthcare administrators, policymakers, and clinicians, Europe can advance trauma care and ensure operating rooms are equipped to meet the demands of the future while delivering optimal outcomes for patients.

## Data Availability

No datasets were generated or analysed during the current study.
